# Pathogenic *Rickettsia*, *Anaplasma*, and *Ehrlichia* in *Rhipicephalus microplus* ticks collected from cattle and laboratory hatched tick larvae

**DOI:** 10.1371/journal.pntd.0011546

**Published:** 2023-08-30

**Authors:** Jiao Xu, Xiao-Lan Gu, Ze-Zheng Jiang, Xiao-Qian Cao, Rui Wang, Qiu-Ming Peng, Ze-Min Li, Li Zhang, Chuan-Min Zhou, Xiang-Rong Qin, Xue-Jie Yu

**Affiliations:** 1 State Key Laboratory of Virology, School of Public Health, Wuhan University, Wuhan City, China; 2 The Second Hospital of Shandong University, Jinan, China; Mahidol Oxford Tropical Medicine Research Unit, THAILAND

## Abstract

**Background:**

The order Rickettsiales contains a group of vector-borne gram-negative obligate intracellular bacteria, which often cause human emerging infectious diseases and economic losses for dairy and meat industries. The purpose of this study is to investigate the distribution of the pathogens including *Rickettsia* spp., *Anaplasma spp*., and *Ehrlichia* spp. in the order Rickettsiales in ticks from Yueyang, a prefecture-level city of Hunan Province in Sothern China, and assess the potentiality of transovarial transmission of these rickettsial organisms.

**Methods:**

Ticks were collected from cattle in a farm in Yueyang City and the tick DNA was used as template to amplify the *htrA*, *rrs*, *gltA*, *ompA* and *ompB* genes of *Rickettsia* as well as *rrs* and *groEL* genes of *Anaplasma* and *Ehrlichia*.

**Results:**

All ticks (465) collected were the cattle tick, *Rhipicephalus microplus*. PCR showed the minimum infection rate (MIR) was 1.5% (7/465) for *Candidatus* Rickettsia xinyangensis, 1.9% (9/465) for *C*. Anaplasma boleense, 1.3% (6/465) for *Anaplasma platys*, 0.6% (3/465) for *A*. *marginale*, and 1.17% (2/465) for each of *A*. *bovis*, *Ehrlichia minasensis*, and a non-classified *Ehrlichia* sp. A human pathogen, *C*. Rickettsia xinyangensis and *A*. *platys* were detected in 100% (3/3) and 33.3% (2/6) laboratory-hatched larval pools from infected females respectively.

**Conclusion:**

Our study revealed a diversity of pathogenic rickettsial species in *R*. *microplus* ticks from Hunan Province suggesting a threat to people and animals in China. This study also provided the first molecular evidence for the potential transovarial transmission of *C*. Rickettsia xinyangensis and *A*. *platys* in *R*. *microplus*, indicating that *R*. *microplus* may act as the host of these two pathogens.

## Author summary

Spotted fever group rickettsiae, *Anapalsma*, and *Ehrlichia* are tick-borne bacteria, which can cause severe human and animal infection and/or extensive economic losses in animal husbandry. It has been well documented that some *Ixodidae* ticks are able to transmit *Rickettsia*, *Anaplasma*, and *Ehrlichia*, while the studies on transovarial transmission are limited. In this study, we detected 7 rickettsial species in the cattle tick *Rhipicephalus microplus* in Hunan Province of the Southern China. Among them, *Candidatus* Rickettsia xinyangensis and *A*. *platys* were also found in the tick eggs and laboratory-hatched larvae of *R*. *microplus*, suggesting the vertical transmission of these two pathogens in *R*. *microplus*. Our findings provide important information regarding the distribution and ecology of these tick-borne rickettsiae.

## Introduction

Ticks are obligate blood-sucking ectoparasites that parasitize on animals and occasionally humans [1]. Ixodidae ticks have been documented to transmit more than 170 pathogens worldwide, including viruses, rickettsiae, spirochetes, and protozoa [2]. These pathogens can result in severe human diseases such as tick-borne forest encephalitis, severe fever with thrombocytopenia syndrome, spotted fever, Lyme disease, and Q fever [2–6]. The cattle tick *Rhipicephalus microplus* in the family Ixodidae is widely distributed worldwide, especially in tropical and subtropical regions [7]. It is regarded as the most economically important ectoparasite of cattle considering the massive economic losses it caused to the dairy and meat industries [8, 9]. Hunan Province in the Southern China has humid and warm weather with little seasonal variation [10]. It is one of the most abundant regions of ticks with *R*. *microplus* as the dominant species [2,10]. The paradox between the limited researches of tick-borne pathogens and the richness of ticks within Hunan suggests further investigation is needed.

The Rickettsiales contains a group of vector-borne gram-negative obligate intracellular bacteria [11]. This order comprises two documented families (Rickettsiaceae and Anaplasmataceae) and one recently established family (*Candidatus* Midichloriaceae) [12]. As well-known zoonotic pathogens, some species in the Rickettsiales can cause severe human diseases and extensive economic losses in animal husbandry [13,14]. In the past 30 years, many novel species of the Rickettsiales have been discovered and the geographic distributions of the Rickettsiales have been expanded dramatically [13,15,16]. Some species of the Rickettsiales were originally considered to be nonpathogenic, but have been found to cause diseases in humans in recent years [13,17–20]. Obviously, the Rickettsiales pose a considerable challenge for public health and require continuous monitoring. Among these tick-borne Rickettsiales, *Rickettsia* spp. [21,22], *Anaplasma* spp. [23–26], and *Ehrlichia* spp. [23,26] are of great concern in Asia and Eurasia.

The pathogen-host relationship shaped by coevolution of the Rickettsiales with arthropods including massive pathogen replication, maintenance of persistent infection, as well as transstadial and transovarial transmission [27]. It has been well documented that some Ixodidae ticks are able to transmit *Rickettsia* spp., *Anaplasma* spp. and *Ehrlichia* spp. transstadially [18,28,29]. Additionally, it is widely accepted that the transovarial transmission plays an important role in the maintenance of *Rickettsia* spp. and *Babesia* spp. [30–33]. Previous studies on transovarial transmission of *Anaplasma* spp. are scarce and controversial on the contrary. Some studies reported *A*. *marginale* and *A*. *centrale* can’t be transmitted transovarially in *R*. *microplus* and *R*. *simus* respectively [34–36], while other studies provided the evidence for transovarial transmission of *A*. *phagocytophilum* in *Dermacentor albipictus*, *A*. *marginale* in *R*. *microplus*, and *A*. *platys* in *R*. *sanguineus* [37–39].

Therefore, we investigated the prevalence of rickettsial species in *R*. *microplus* collected from cattle in Hunan Province and determined whether the rickettsial organisms existed in tick eggs and laboratory hatched larvae.

## Material and methods

### Ethical statement

The collection of ticks for molecular detection was approved by the Ethics Committee of the Medical School, Wuhan University (WHU2020-YF0023), and any possible efforts were made to minimize the pain of animals.

### Tick collection

**Fig 1:**
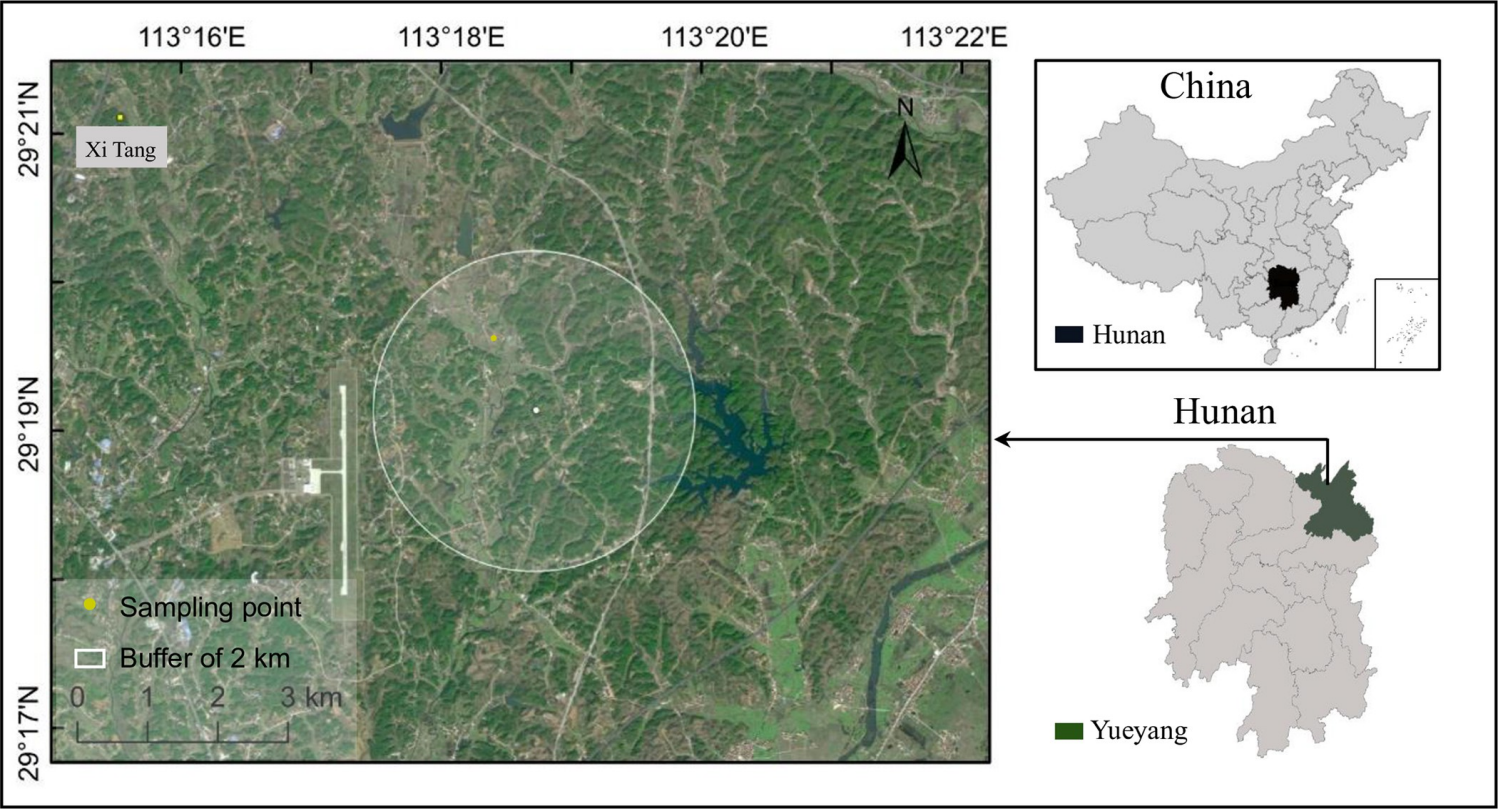
Map of sampling site. Ticks were collected from cattle in a farm in Xi Tang Town, Yueyang City, Hunan Province in Southern China from August 5 to August 22, 2022. The map was constructed using ArcGIS 10.6 software. The basemap shapefiles were downloaded from national platform for common geospatial information services (tianditu.gov.cn).

Ticks were collected from a farm with 50 free-range cattle in Yueyang City, a prefecture city in Hunan Province in Southern China from August 5 to August 22, 2022 (Fig 1). There was a large free-range hilly area (approximately 2 km in radius) (Fig 1), which facilitated the survival of ticks and tick bites on cattle. To ensure the viability of the ticks, each tick was carefully held the head with a fine-tipped tweezer and was dragged out in the direction parallel to the host skin [40]. The collected ticks were placed in 5 ml centrifuge tubes with a triangular-shaped ventilation hole on the cover. Each pool was labeled and recorded with corresponding information. After sampling, all ticks were kept in an incubator with 90% relative humidity at room temperature and immediately transported back to our laboratory [41].

**Fig 2:**
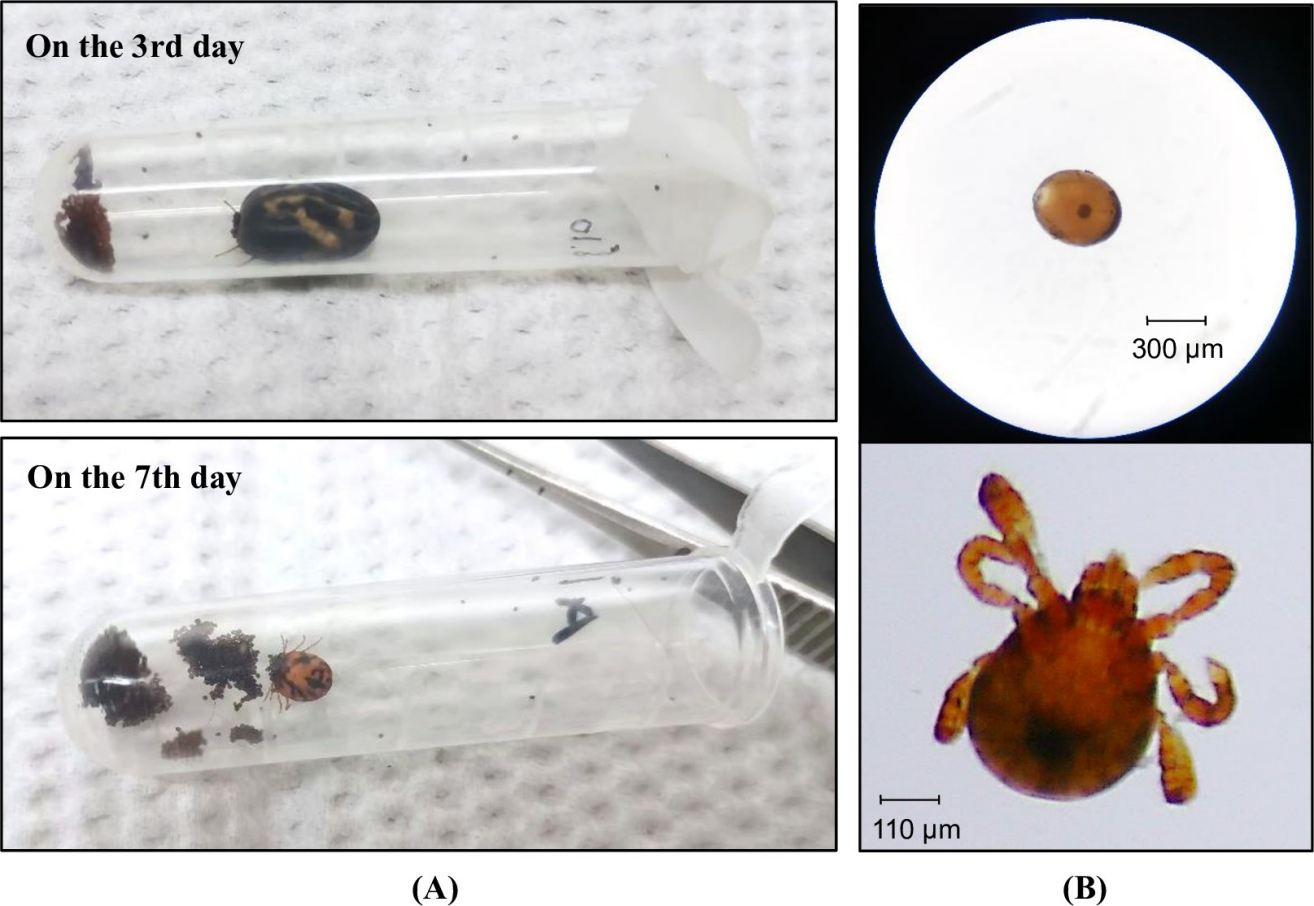
The oviposition of a female *Rhipicephalus microplus*. (A) Photographs taken on the 3rd and 7th day of oviposition, respectively. (B) Micrographs of tick eggs (top) and laboratory hatched larvae (bottom).

Upon arriving at the laboratory, the fully engorged female ticks were placed in a tube individually and they oviposited in a week (Fig 2). The eggs (n = 100) of each tick were used for DNA extraction and the remaining eggs were incubated to hatch. For DNA extraction, the non-engorged or partially engorged ticks were pooled according to the sampling date, sex (adults only), and life stage [42]; the fully engorged female ticks were used individually after oviposition; the eggs (n = 100) or larvae (n = 100) from each tick were used in one pool. All ticks were stored at -80°C before DNA extraction.

### DNA extraction

Prior to DNA extraction, samples were rinsed with 75% ethanol for 5min, then washed and soaked with pure water to remove surface impurities and residual ethanol. The samples in the 2-ml Eppendorf tubes were immersed in liquid nitrogen to make the specimen brittle. One magnetic bead with 200 μl deionized water, 200 μl Buffer gA1 and 20 μl Proteinase K was added to each tube. Samples were ground with a frozen mixed grinding apparatus (Retsch, Germany) for 10min and centrifuged at 12,000 g for 5min. DNA was extracted from supernatant with a Trelief Animal Genomic DNA Kit (Tsingke Biotechnology, Beijing, China) and stored at -80°C.

### Molecular detection of *Rickettsia* spp., *Anaplasma* spp. and *Ehrlichia* spp

The tick DNA was used as template for PCR amplification of *Rickettsia* spp., *Anaplasma* spp., and *Ehrlichia* spp. with primers listed in Table 1. The PCR reactions were performed in a 15 μl mixture containing 7.5 μl 2X Taq Master Mix (TaKaRa, Shiga, Japan), 1.5 μl 10 μM each forward and reverse primer (Sangon Biotech, Shanghai, China), 2.5 μl nuclease-free water, and 2 μl sample DNA. Nuclease-free water was used as negative control in each PCR reaction. The PCR protocol included an initial denaturation at 95°C for 5min followed by 35 cycles of denaturation at 95°C (30s), annealing at 50–57°C (30s) (Table 1), extension at 72°C (30s–60s), and an additionally final extension at 72°C for 10min.

**Table 1:** PCR primers used to amplify *Rickettsia*, *Anaplasma* and *Ehrlichia* species.

Target species	Genes	Primer names	Sequences	Annealing temperature (°C)	PCR product sizes		References
Tick	*rrs*	16S-F	AGTATTTTGACTATACAAAGGTATTG	50			
		16S-R	GTAGGATTTTAAAAGTTGAACAAACTT			408 bp	[43]
*Rickettsia*	*rrs*	S1	TGATCCTGGCTCAGAACGAAC	57			
		S2	TAAGGAGGTAATCCAGCCGC				
		S3	AACACATGCAAGTCGRACGG	57			
		S4	GGCTGCCTCTTGCGTTAGCT			1,317 bp	[44]
*Rickettsia*	*gltA*	*gltA*1	TGATCCTGGCTCAGAACGAAC	50			
		*gltA*2	TAAGGAGGTAATCCAGCCGC				
		*gltA*3	AACACATGCAAGTCGRACGG	50			
		*gltA*4	GGCTGCCTCTTGCGTTAGCT			667 bp	[45]
*Rickettsia*	*htrA*	17kd5	GCTTTACAAAATTCTAAAAACCATATA	52			
		17kd3	TGTCTATCAATTCACAACTTGCC				
		17kd1	GCTCTTGCAACTTCTATGTT	55			
		17kd2	CATTGTTCGTCAGGTTGGCG			434 bp	[46]
*Rickettsia*	*ompB*	OF	GTAACCGGAAGTAATCGTTTCGTAA	52			
		OR	GCTTTATAACCAGCTAAACCACC				
		SFG IF	GTTTAATACGTGCTGCTAACCAA	55			
		SFG IR	GGTTTGGCCCATATACCATAAG			418 bp	[47]
*Rickettsia*	*ompA*	Rr190.70p	ATGGCGAATATTTCTCCAAAA	50			
		Rr190.701n	GTTCCGTTAATGGCAGCATCT			631 bp	[48]
*Anaplasma/ Ehrlichia*	*rrs*	EC9	TACCTTGTTACGACTT	50			
		EC12A	TGATCCTGGCTCAGAACGAACG				
		EM87F	GGTTCGCTATTAGTGGCAGA	52			
		EM584R	CAGTATTAAAAGCCGCTCCA			477 bp	[49]
*A*. *platys*	*groEL*	P-F1	AGTCGATTAGGGAAGTAGTAC	50			
		P-F2	AGGATGGCTACAAGGTAATG	52			
		P-R	GCGTCCTCTACTCTGTCTT			853 bp	[50]
*A*. *marginale*	*groEL*	M-F1	ACATGCTCCATACTGACTGC	54			
		M-F2	AGATGAGATTGCACAGGTTG	52			
		M-R	AGATGCAAGCGTGTATAGCAG			741 bp	[50]
*C*. Anaplasma boleense	*groEL*	CA-F1	TAGAAGACGCGGTAGGCT	52			
		CA-F2	GTACTGCAGGCCCTAAAG	52			
		CA-R	AACGTTCTCCAATATGGGAAG			547 bp	[50]
*Ehrlichia*	*groEL*	E-F1	GAAGA TGCTGTAGGRTGTACDGC	57			
		E-F2	ATTRCTCARAGTGCTTCHCARTG	55			
		E-R	AGHGCTTCWCCTTCYACATCYTC			537 bp	[50]

Note: *A*. = *Anplasma*, *C*. = *Candidatus*

### Sequencing and phylogenetic analysis

PCR products were electrophoresed on 1.0% gel and DNA bands with expected size were excised from gels and purified with a gel extraction kit (Tsingke Biotechnology, Beijing, China). The purified DNA was cloned into the pMD19-T vector (TaKaRa, Beijing, China) and transformed into *Escherichia coli* DH5α competent cells. Positive clones were sequenced (Sangon, Shanghai, China). The chromatograms of DNA sequences were analyzed with Chromas (Technelysium, Tewantin, QLD, Australia) for accuracy. The Nucleotide Basic Local Alignment Search Tool (BLASTn) (https://blast.ncbi.nlm.nih.gov/Blast.cgi) was applied to compare the sequences from this study with those in the GenBank for species identification. Sequences of interest were imported into Molecular Evolutionary Genetics Analysis (MEGA) software (version 7.0) for alignment and editing. Phylogenetic trees were constructed based on the Maximum Likelihood (ML) method with the Kimura 2-parameter model, and bootstrap values were inferred from 1,000 replicates [44].

## Results

### Tick samples

**Fig 3:**
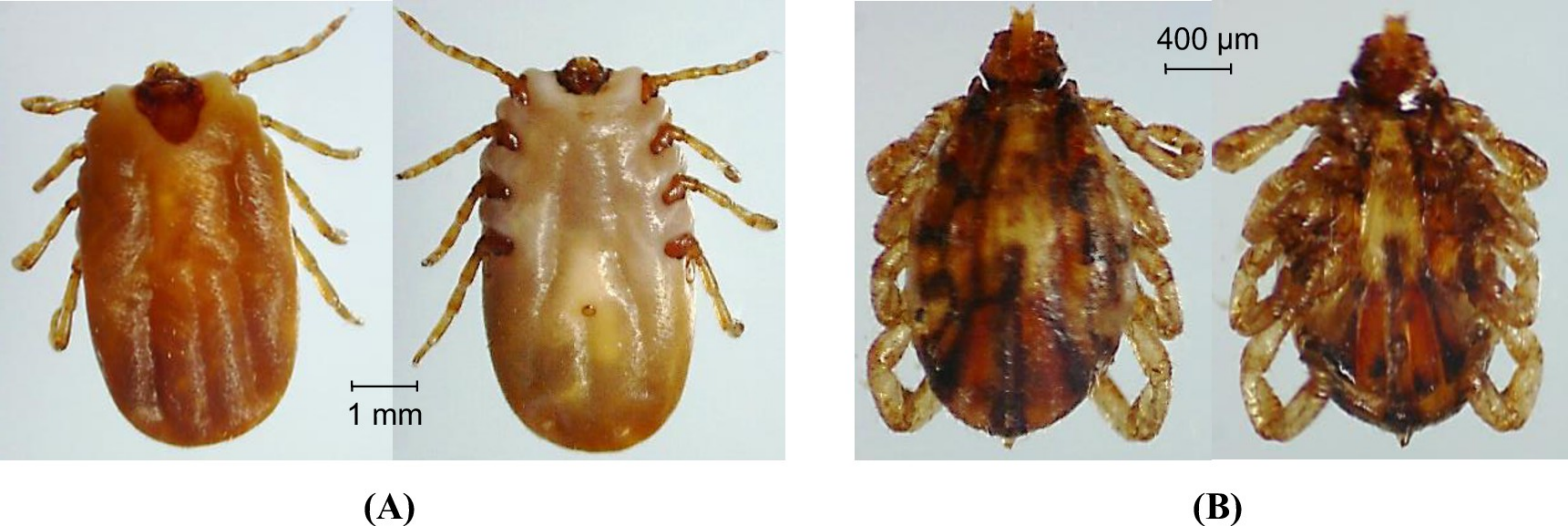
Photos of the dorsal (left) and ventral (right) sides of *Rhipicephalus microplus* under a dissecting microscope. (A) An adult female tick. (B) An adult male tick.

**Fig 4:**
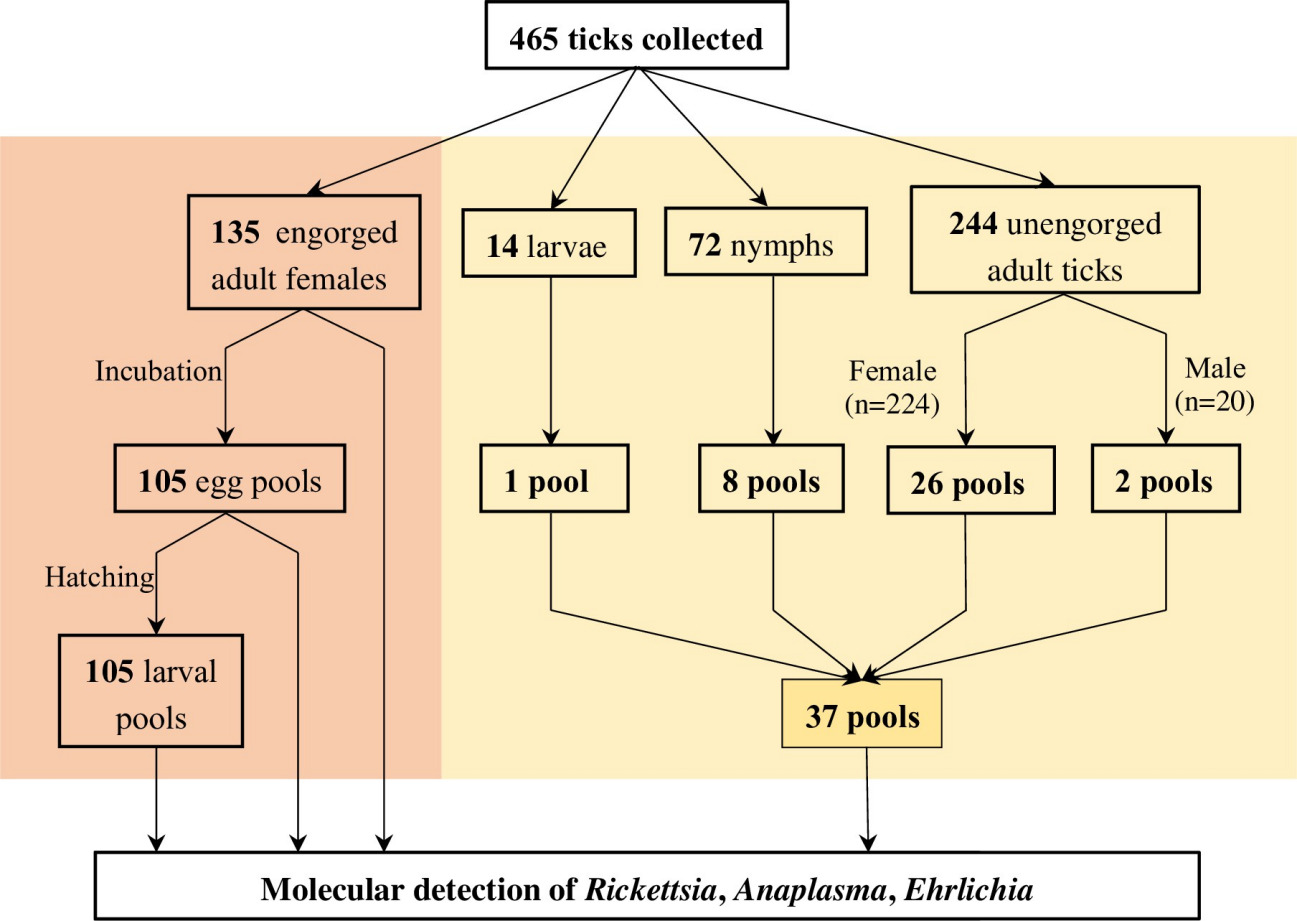
Sample pooling information.

In total, 465 ticks were collected, including 379 adults, 72 nymphs, and 14 larvae. The adult ticks contained male ticks (n = 20), fully engorged female ticks (n = 135), and non-fully-engorged females (n = 224) (Fig 3). All samples were divided into two parts: fully engorged adult females (n = 135) were used separately and the rest (n = 330) were divided into 37 pools (Fig 4). Of 135 engorged adult female ticks, 105 (78%) of them laid eggs in the laboratory and the mass of egg clutches ranged from 61 to 374 mg. All 105 egg clutches hatched to larvae (Fig 2).

**Fig 5:**
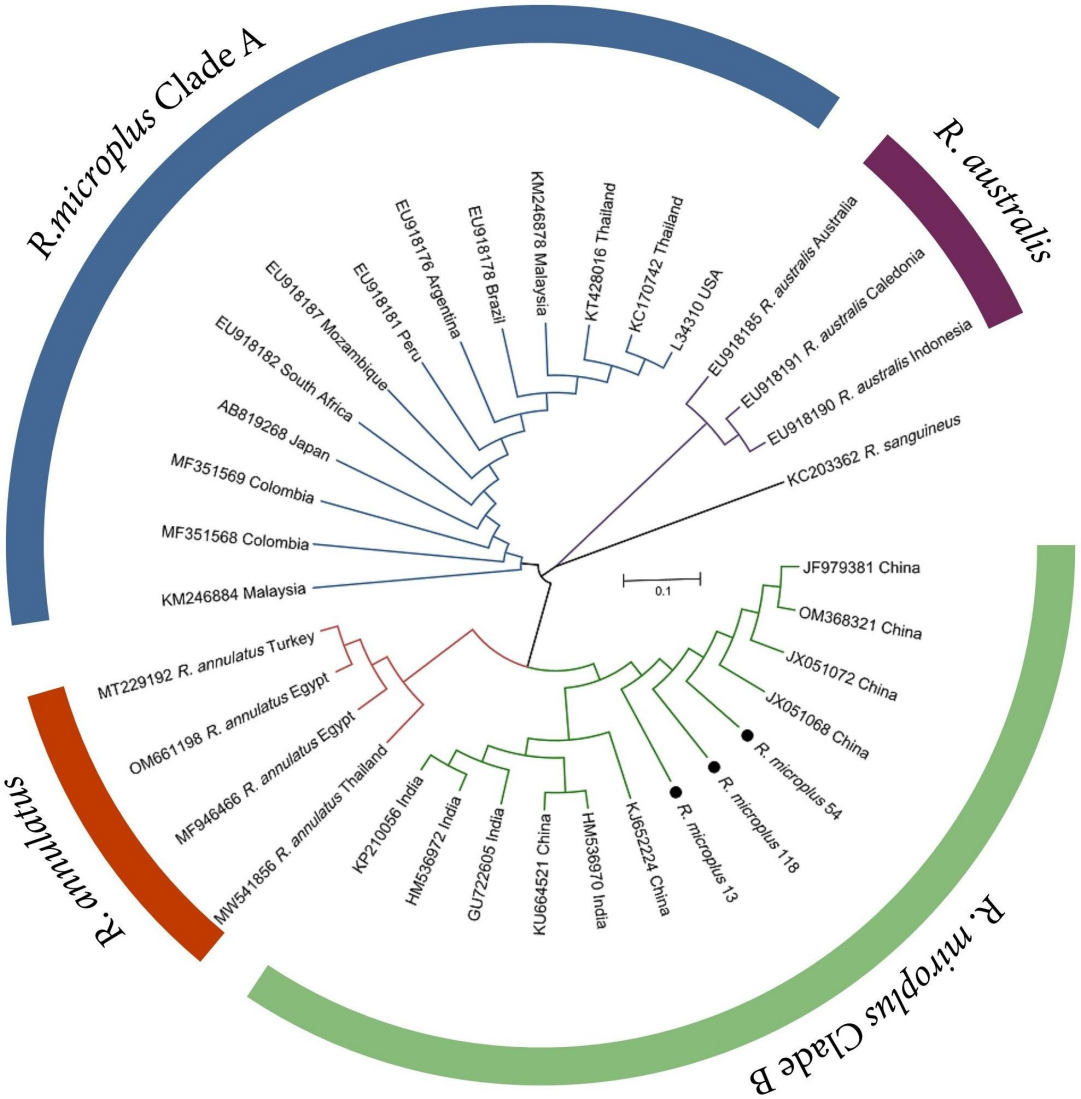
Phylogenetic tree of *Rhipicephalus microplus* ticks. The tree was constructed using the partial 16S rRNA gene (229 bp) of ticks based on the Maximum Likelihood (ML) method with the Kimura 2-parameter model in MEGA 7.0. *Rhipicephalus sanguineus* sequence was used for outgroup in the tree. The *R*. *microplus* sequences obtained in this study were marked with dots and have been submitted to the GenBank with accession numbers: OQ975295–OQ975297.

All ticks were preliminary morphologically identified as *R*. *microplus* and then molecularly confirmed by amplification and sequencing of endogenous tick 16S rRNA gene (Fig 3 and Table 1). They had a dentition in the typical 4/4 column arrangement and a palpal article 1 ventrally lacking a ventrointernal protuberance bearing setae [42, 51, 52]. Male *R*. *microplus* ticks carried a typical caudal appendage on the ventral plates [42] (Fig 3). The partial 16S rRNA gene sequences obtained from these samples shared 100% identities with those of *R*. *microplus* in the GenBank. Phylogenetic analysis indicated that *R*. *microplus* of this study were clustered with those from China and India in the *R*. *microplus* clade B sensu [53], which was sister to *R*. *annulatus* (Fig 5). *R*. *microplus* from Africa, Americas and Southeast Asia formed *R*. *microplus* clade A sensu [53]. *R*. *annulatus*, *R*. *australis* (formerly *R*. *microplus*) and two clades of *R*. *microplus* constitute *R*. *microplus* species complex (Fig 5).

### Detection of Rickettsiales species in ticks

One *Rickettsia* species was identified with PCR in the ticks with a minimum infection rate (MIR) at 1.5% (7/465) (Table 2). The MIR was calculated by assuming only one tick was infected in a positive pool [45]. *Rickettsia* positive samples comprised 3 engorged adult females, 1 larval pool, 1 nymphal pool, and 2 non-fully-engorged adult female pools (S2 Dataset). DNA sequencing showed that the *Rickettsia* from the ticks was most closely related to *C*. Rickettsia xinyangensis, which was firstly detected from patients in central China [54]. The highest identities between DNA sequences from the *R*. *microplus* ticks and those of *C*. Rickettsia xinyangensis were all 100% on *htrA*, *rrs*, *ompA*, *gltA* and *ompB* genes (Table 3). Among these known identical sequences of *C*. Rickettsia xinyangensis, *ompA* gene sequence (KU853021) and *gltA* gene sequence (KU853023) were amplified from patients, while *htrA* gene sequence (KY617773), *rrs* gene sequence (KY617772), and *ompB* gene sequence (KY617776) were obtained from *Haemaphysalis longicornis* ticks around the patients’ residences.

**Table 2:** Prevalence of *Rickettsia*, *Anaplasma*, and *Ehrlichia* in *Rhipicephalus microplus* ticks collected from cattle in Hunan Province, China from August 5 to August 22, 2022.

Pathogens	Overall minimum infection rate (n = 465)	Positive engorged adult females/Positive rate (n = 135)	Positive tick pools/Positive rate (n = 37)
*C*. Rickettsia xinyangensis	1.5%	3/2.2%	4/10.8%
*A*. *platys*	1.3%	6/4.4%	0
*A*. *marginale*	0.6%	1/0.7%	2/5.4%
*A*. *bovis*	0.4%	1/0.7%	1/2.7%
*C*. Anaplasma boleense	1.9%	5/3.7%	4/10.8%
*E*. *minasensis*	0.4%	0	2/5.4%
Non-classified *Ehrlichia* sp.	0.4%	1/0.7%	1/2.7%

Note: *C*. = *Candidatus*, *A*. = *Anplasma*, *E*. *= Ehrlichia*.

**Table 3:** The rickettsial sequences in the GenBank sharing the highest homology with the rickettsial sequences obtained in this study.

Species	Known gene sequences with highest identity (Identity rate)				
	*gltA* (604 bp)	*rrs* (1241 bp)	*ompA* (325 bp)	*ompB* (344 bp)	*htrA* (365 bp)
*C*. Rickettsia xinyangensis	KU853023 (100%)	KY617772 (100%)	KU853021 (100%)	KY617776 (100%)	KY617773 (100%)
*C*. Rickettsia longicornii	MN026549 (100%)	MT747412 (99.76%)	MN026548 (100%)	MT747415 (100%)	MG906673 (100%)
Novel species criteria [55]	<99.9%	<99.8%	<98.8%	<99.2%	-

Note: *C*. = *Candidatus*.

Four *Anaplasma* species were amplified from the ticks with PCR. Six engorged female adults were PCR positive for *A*. *platys*. The MIR for *A*. *platys* in the ticks was 1.3% (6/465) (Table 2). The sequence homology between the *A*. *platys* in this study and those in the GenBank was 100% for *rrs* and 84.22%–100% for *groEL*. The sequences of *A*. *marginale* were obtained from 1 engorged female adult and 2 non-fully-engorged adult female pools (S2 Dataset). The MIR for *A*. *marginale* was 0.6% (3/465) in the ticks and the sequences from the ticks were 100% identical on both *rrs* and *groEL* genes with *A*. *marginale* sequences in the database. One engorged female adult and one tick pools containing 6 non-fully-engorged adult females were positive for *A*. *bovis*. The MIR for *A*. *bovis* was 0.4% (2/465, 1.17%) in the ticks. The sequences from the ticks shared 100% homology to those of *A*. *bovis* in the GenBank on *rrs* gene. *C*. Anaplasma boleense positive samples comprised 5 engorged female ticks and 4 non-fully-engorged female adult pools (S2 Dataset). The MIR for *C*. Anaplasma boleense sequences was 1.9% (9/465) in the ticks and the sequences of *rrs* and *groEL* genes from the ticks shared 99.30%–99.79% and 96.80%–100% homology, respectively with sequences of *C*. Anaplasma boleense in the GenBank.

Two *Ehrlichia* species were amplified from the ticks. One *Ehrlichia* species (2/465, 0.4%), detected in 1 nymphal pool and 1 non-fully-engorged adult female pool (S2 Dataset), was closely related to *E*. *minasensis* with the identities of 100% for *rrs* and 99.35%–100% for *groEL*. Another *Ehrlichia* species, detected in 1 engorged adult female and 1 non-fully-engorged adult female pool, was related to *Ehrlichia* sp. Dehong-17 (OL838197 and OL907298) with the homology of 100% for *rrs* and 99.78%–100% for *groEL*.

Among 3 *C*. Rickettsia xinyangensis positive female ticks, all their egg clutches and laboratory-hatched larvae were also positive for *C*. Rickettsia xinyangensis; among 6 *A*. *platys* positive female ticks, 3 ticks’ egg clutches and 2 ticks’ larvae were also positive for *A*. *platys*. The DNA of *A*. *marginale*, *A*. *bovis*, *C*. Anaplasma boleense, *E*. *minasensis*, and a non-classified *Ehrlichia* sp. was not found in tick eggs or laboratory-hatched larvae.

The representative DNA sequences of rickettsial organisms obtained in this study have been submitted to the GenBank with accession numbers: OQ506629–OQ506639, OQ509025–OQ509032, and OR062291–OR062294.

### Phylogenetic analysis

**Fig 6:**
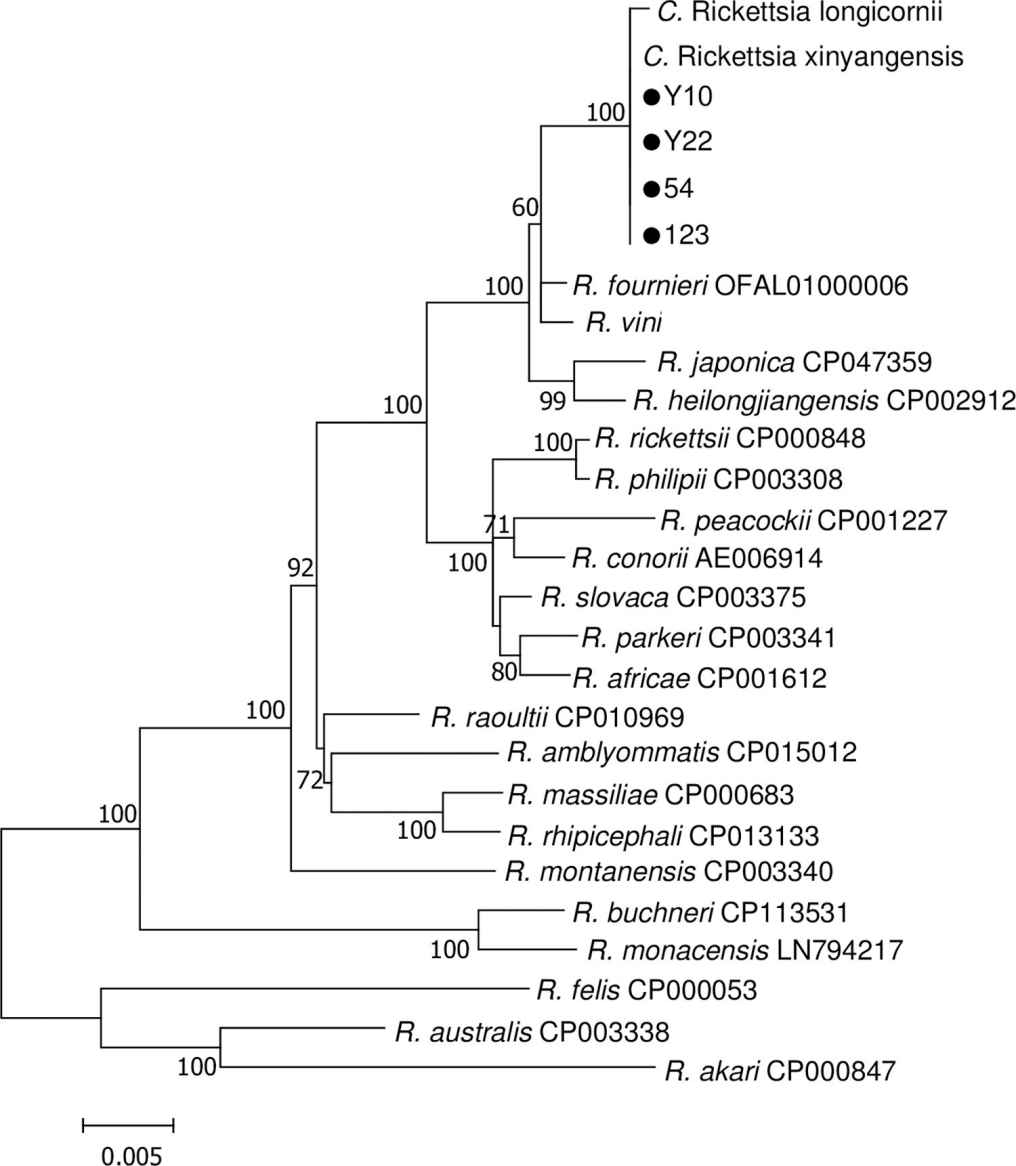
Phylogenetic tree of *Rickettsia* based on the concatenated sequences of *htrA* (365 bp), *rrs* (1241 bp), *ompA* (551 bp), *gltA* (604 bp), and *ompB* (344 bp) genes. Phylogenetic tree was constructed based on the Maximum Likelihood (ML) method with the Kimura 2-parameter model in MEGA 7.0. Bootstrap values (inferred from 1,000 replicates) >60% were indicated. The *Rickettsia* sequences obtained in this study were marked with dots. For the *Rickettsia* species without complete genome sequences, the GenBank accession nos. in the order of *htrA*, *rrs*, *ompA*, *gltA* and *ompB* are KY617773, KY617772, KU853021, KU853023, and KY617776 for *C*. Rickettsia xinyangensis; MG906673, MT747412, MN026548, MN026549, and MT747415 for *C*. Rickettsia longicornii; KT187396, MT062904, KT326194, KT187394, and JF758826 for *Rickettsia vini*.

Phylogenetic analysis based on the concatenated sequences of *htrA*, *rrs*, *ompA*, *gltA* and *ompB* genes showed that the *Rickettsia* strain from 4 *R*. *microplus* ticks was identical with *C*. Rickettsia xinyangensis and formed a monoclade with *C*. Rickettsia xinyangensis and *C*. Rickettsia longicornii (Fig 6).

**Fig 7:**
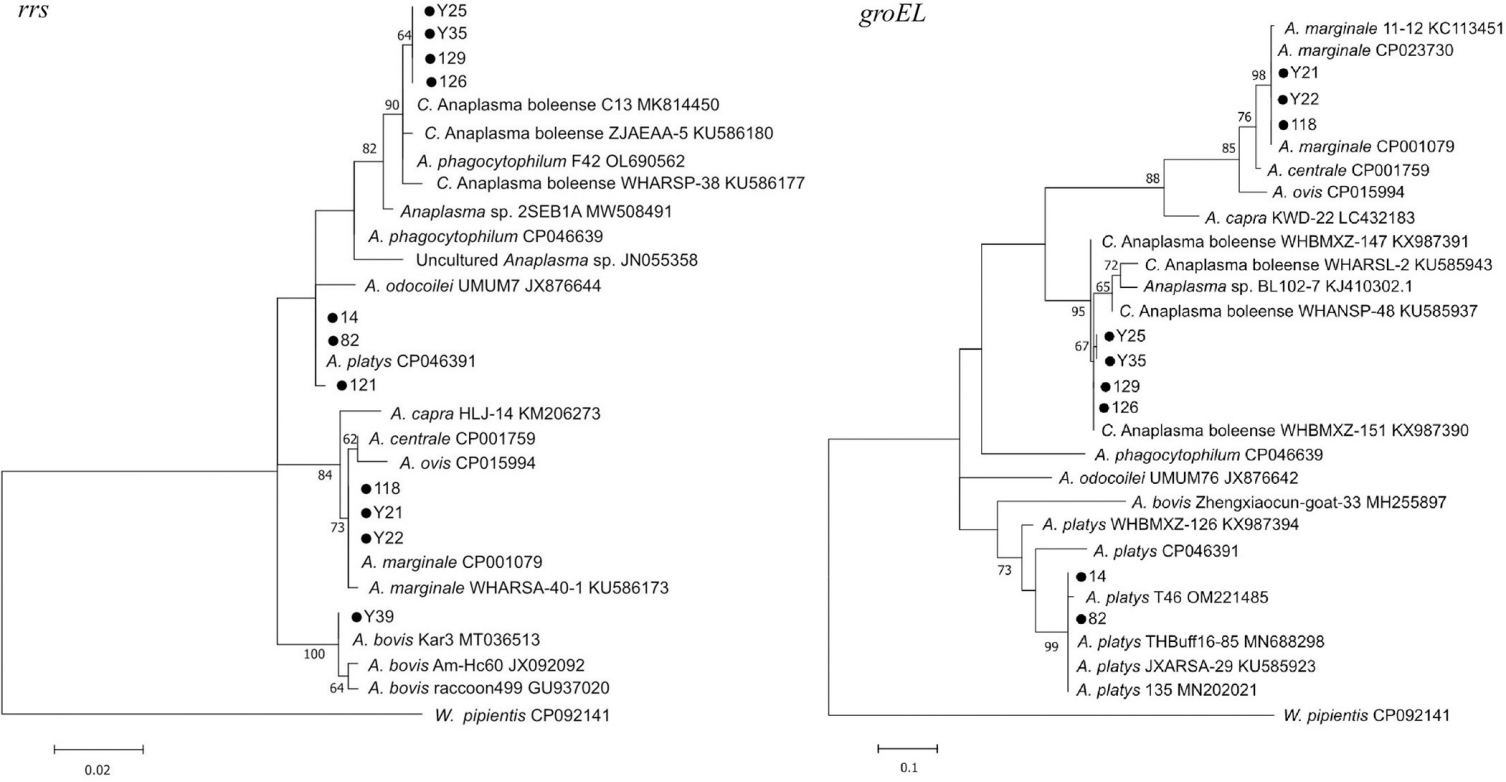
Phylogenetic trees of *Anaplasma* based on the partial *rrs* (470 bp) gene and *groEL* (205 bp) genes. Phylogenetic trees were constructed based on the Maximum Likelihood (ML) method with the Kimura 2-parameter model in MEGA 7.0. Bootstrap values (inferred from 1,000 replicates) more than 60% were indicated. *Wolbachia pipientis* sequences were used for outgroup in the trees. The *Anaplasma* sequences obtained in this study were marked with dots.

Phylogenetic analysis indicated that the *Anaplasma* sequences amplified from ticks were divided into 4 different clusters in the phylogenetic trees (Fig 7). Phylogenetic trees of both *rrs* and *groEL* sequences showed that 3 clones (Y21, Y22 and 118) were clustered together with *A*. *marginale*; 3 clones (14, 82, and 121) were clustered together with *A*. *platys*; 4 clones (Y25, Y35, 126 and 129) were clustered in the same clade with *C*. Anaplasma boleense (Fig 7). One clone (Y39) was clustered in the same clade with *A*. *bovis* with *rrs* gene sequence, but we were unable to get the PCR product of the *groEL* gene from the ticks.

**Fig 8:**
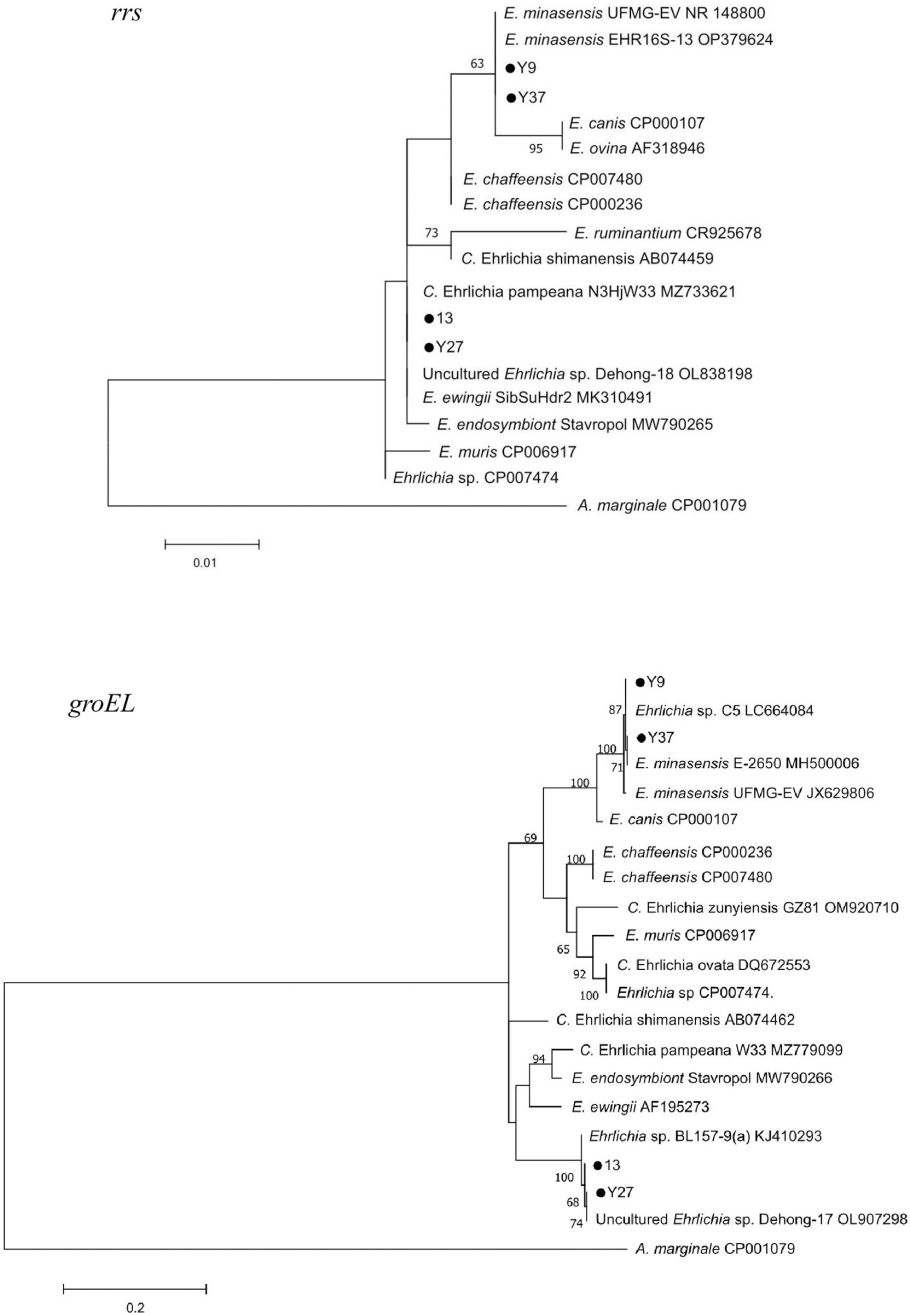
Phylogenetic trees of *Ehrlichia* with the partial *rrs* (430 bp) and *groEL* (461 bp) genes. Phylogenetic trees were constructed using the Maximum Likelihood (ML) method with the Kimura 2-parameter model in MEGA 7.0. Bootstrap values (inferred from 1,000 replicates) more than 60% were indicated. *Anaplasma marginale* sequences were used for outgroup in the trees. The *Ehrlichia* sequences obtained in this study were marked with dots.

Phylogenetic analysis with the *rrs* and *groEL* gene sequences showed that the *Ehrlichia* sequences from the ticks were divided into two different clusters (Fig 8). One group (Y9 and Y37) were clustered together with *E*. *minasensis* from *R*. *microplus* from Thailand (OP379624), Brazil (NR_148800 and JX629806) and Australia (MH500006); another group (13 and Y27) were closely related to *Ehrlichia* sp. Dehong-17, which was amplified from *R*. *microplus* in Yunnan Province, Southwestern China.

## Discussion

In this study, we detected the DNA of 1 spotted fever group (SFG) rickettsiae, 4 *Anaplasma* species and 2 *Ehrlichia* species in the cattle tick *R*. *microplus* collected from cattle in a farm in Hunan Province of the Southern China, revealing a high diversity of rickettsial organisms in *R*. *microplus*. To our knowledge, only two of these seven species have been previously reported in Hunan Province [10], so this study contributes to a better understanding of the distribution of these rickettsial organisms.

Among seven rickettsial organisms, the DNA of *C*. Rickettsia xinyangensis and *A*. *platys* has been previously detected in patients [54,56]. Additionally, *A*. *marginale* and *A*. b*ovis* are known to be agent of bovine anaplasmosis, while *A*. *platys* and *E*. *minasensis* are the causative agents of canine infectious cyclic thrombocytopenia (ICCT/ CICT) and bovine ehrlichiosis, respectively. They may affect animal health and livestock husbandry though this study didn’t report the infection status of animal hosts. The tick pools positive for rickettsial organisms were collected from a same herd on different dates. One possible explanation is that the cattle were infected with rickettsial organisms and the ticks got infected when they parasitized on cattle and sucked the blood; another explanation is that there were natural foci of these rickettsial organisms in the field. The application of cattle vaccines and regular cleaning of parasitic ticks are of great necessity, especially for free-range livestock keepers.

*Candidatus* Rickettsia xinyangensis is a novel uncultured *Rickettsia* species identified in patients in the Central China [54]. The patients had a mild fever, leukopenia, elevated hepatic enzyme levels and eschars on the body. *Haemaphysalis longicornis* ticks collected around the 3 patients’ residences were also positive for *C*. Rickettsia xinyangensis suggesting their possible involvement in transmission [54]. We detected *C*. Rickettsia xinyangensis in the tick eggs and laboratory-hatched larvae of *R*. *microplus*, indicating that *C*. Rickettsia xinyangensis can be transovarially transmitted in *R*. *microplus* tick. Transovarial transmission of *C*. Rickettsia xinyangensis has not yet reported in other tick species. Other *Rickettsia* species such as *Rickettsia africae* in *Amblyomma variegatum* [32], *Rickettsia parkeri* in *Amblyomma maculatum* and *R*. *microplus* [57,58], *Rickettsia rickettsii* in *Amblyomma aureolatum* [28], *Rickettsia bellii* in *Ixodes loricatus* [29], and *Rickettsia parkeri* strain Atlantic rainforest in *Amblyomma ovale* [30], have been demonstrated to be transovarially transmitted. The transovarial transmission (TOT) rate, proportion of infected females giving rise to at least one positive egg or larva [59], could be up to 100% in controlled laboratory settings [28,32,59].

*Anaplasma platys* was thought to be a canine pathogen causing a chronic infection with weight loss, anorexia, fever, lethargy, and lymphadenomegaly [60,61]. It has also been reported to cause human infection with nonspecific clinical symptoms, including headache and muscle pains [56]. *Anaplasma platys* sequences detected in *R*. *microplus* in this study were clustered with those from cattle (MN202021), buffalo (MN688298), and mosquitoes (KU585923), showing its wide host tropism. Unlike *Rickettsia* spp., transovarial transmission of most *Anaplasma* spp. and *Ehrlichia* spp. was thought to occur at low frequencies or not at all [36,62]. A previous study have showed that transovarial transmission of *A*. *phagocytophilum* in *Dermacentor albipictus* was at the efficiency of 10%–40% [38]. In this study, we showed the transovarial transmission of *A*. *platys* in the *R*. *microplus* at the rate of 33.3% from the infected females to laboratory-hatched larvae, suggesting that *R*. *microplus* may act as the host of *A*. *platys*. A previous study reported vertical transmission of *A*. *platys* in *R*. *sanguineus* under controlled laboratory conditions [37].

*Ehrlichia minasensis* is a novel pathogen causing fever, lethargy, thrombocytopenia and depression in bovines [63]. It was first identified in cattle from Canada [64] and later in Brazil [65] and Colombia [66]. Besides the American continent, it has also been detected in France [65], Ethiopia [67], South Africa [68], Pakistan [69] and Israel [70]. In China, there were only two reports of *E*. *minasensis*, one detected *E*. *minasensis* in *Haemaphysalis hystricis* ticks from Hainan Island [71] and the other recently found this pathogen in *R*. *microplus* from Guizhou Province [72]. Our study first reported the *E*. *minasensis* infection in *R*. *microplus* in Hunan Province, China. Since the evidence of transstadial transmission of *E*. *minasensis* in *R*. *microplus* was reported [73], the role of *R*. *microplus* in the transmission of this pathogen should be highly valued [74].

One limitation of our study is the small sample size of ticks and another is that we tested tick eggs and laboratory hatched larvae in pools rather than individually. Both limitations may cause bias on the prevalence and TOT rate of *Rickettsia* and *Anaplasma* in the ticks.

## Conclusion

Our study revealed a diversity of pathogenic rickettsial species in *R*. *microplus* ticks from Hunan Province suggesting a threat to people and animals in China. This study also provided the first molecular evidence for the potential transovarial transmission of *C*. Rickettsia xinyangensis and *A*. *platys* in *R*. *microplus*, indicating that *R*. *microplus* may act as the host of these two pathogens.

## Supporting information

S1 Dataset Host data of positive engorged female adult ticks. (PDF)

S2 Dataset Tick pool data. (PDF)
