# Supplementary material for: Pathogenic Rickettsia, Anaplasma, and Ehrlichia in Rhipicephalus microplus ticks collected from cattle and laboratory hatched tick larvae
Source: PLoS Negl Trop Dis. 2023 Aug 30;17(8):e0011546. doi: 10.1371/journal.pntd.0011546 (PMC10468208; doi:10.1371/journal.pntd.0011546)
Supplement: S1 Dataset — (PDF) [file pntd.0011546.s001.pdf]

Pathogens	Positive engorged adult female No.	Host No.
C. Rickettsia jingxinensis	54**	20
	123**	41
	131**	47
A. platys	7	4
	14**	6
	59	22
	82**	29
	103	35
	135*	50
A. marginale	118	40
A. bovis	132	49
C. Anaplasma boleense	27	11
	44	18
	46	18
	126	44
	134	50
Non-classified *Ehrlichia* sp.	13	6

Note: *C.= Candidatus, A.=Anplasma*, *E.= Ehrlichia*. "*" indicates that the female tick's corresponding eggs were also positive, "**" indicates that the female tick's corresponding eggs and larvae were both positive.
