# Supplementary material for: Pathogenic Rickettsia, Anaplasma, and Ehrlichia in Rhipicephalus microplus ticks collected from cattle and laboratory hatched tick larvae
Source: PLoS Negl Trop Dis. 2023 Aug 30;17(8):e0011546. doi: 10.1371/journal.pntd.0011546 (PMC10468208; doi:10.1371/journal.pntd.0011546)
Supplement: S2 Dataset — (PDF) [file pntd.0011546.s002.pdf]

Collection date	Pool No.	Life stage	Sex	No. of ticks	Pathogens
8.5	1	adult	female	7	*C.* Anaplasma boleense
8.6	2	adult	female	10	-
	3	adult	female	10	-
	4	adult	female	10	*C.* Anaplasma boleense
	5	adult	male	10	-
	6	adult	male	10	-
	7	nymph	-	10	-
	8	nymph	-	10	-
	9	nymph	-	10	-
	10	nymph	-	8	*E. minasensis*
	11	nymph	-	7	*C.* Rickettsia jingxinensis
8.10	12	adult	female	6	-
	13	adult	female	6	-
	14	adult	female	7	-
	15	adult	female	10	-
	16	adult	female	10	-
	17	adult	female	10	-
	18	adult	female	10	*A. marginale*
	19	adult	female	10	*C.* Rickettsia jingxinensis, *A. marginale*
	20	adult	female	10	-
	21	adult	female	10	-
	22	adult	female	10	*C.* Anaplasma boleense
	23	adult	female	10	-
	24	adult	female	10	Non-classified *Ehrlichia* sp.
8.11	25	adult	female	10	-
	26	adult	female	10	*C.* Rickettsia jingxinensis
	27	adult	female	10	-
	28	nymph	-	10	-
	29	nymph	-	10	-
	30	nymph	-	7	-
	31	larvae	-	14	*C.* Rickettsia jingxinensis
8.12	32	adult	female	5	*C.* Anaplasma boleense
	33	adult	female	8	-
	34	adult	female	7	*E. minasensis*
8.22	35	adult	female	6	*A. bovis*
	36	adult	female	6	-
	37	adult	female	6	-
Total				330	

Note: *C.= Candidatus, A.=Anplasma*, *E.= Ehrlichia*.
